# Anatomical and morphometric evaluation of the orbit, eye tunics, eyelids and orbital glands of the captive females of the South African painted dog (*Lycaon pictus pictus* Temminck, 1820) (Caniformia: Canidae)

**DOI:** 10.1371/journal.pone.0249368

**Published:** 2021-04-19

**Authors:** Wojciech Paszta, Joanna E. Klećkowska-Nawrot, Karolina Goździewska-Harłajczuk

**Affiliations:** 1 Wroclaw Zoological Garden, Wrocław, Poland; 2 Department of Biostructure and Animal Physiology, Faculty of Veterinary Medicine, Wrocław University of Environmental and Life Sciences, Wroclaw, Poland; Faculty of Animal Sciences and Food Engineering, University of São Paulo, BRAZIL

## Abstract

In this study, we present the first data concerning the anatomical, morphometrical, histological and histochemical study of the orbit, eye tunics, eyelids and orbital glands in South African Painted Dogs (*Lycaon pictus pictus*). The study was performed using eyeball morphometry, analysis of the bony orbit including its morphometry, macroscopic study, morphometry, histological examination of the eye tunics and chosen accessory organs of the eye and histochemical analysis. The orbit was funnel shaped and was open-type. There was a single ethmoid opening for the ethmoid nerve on the orbital lamina. The pupil was round, while the ciliary body occupied a relatively wide zone. The iris was brown and retina had a pigmented area. The cellular *tapetum lucidum* was semi-circular and milky and was composed of 14–17 layers of tapetal cells arranged in a bricklike structure. In the lower eyelid, there was a single conjunctival lymph nodule aggregate. One or two additional large conjunctval folds were observed within the posterior surface of the upper eyelids. The superficial gland of the third eyelid had a serous nature. The third eyelid was T-shaped and was composed of hyaline tissue. Two to three conjunctival lymph nodul aggregates were present within the bulbar conjunctiva of the third eyelid. The lacrimal gland produced a sero-mucous secretion. A detailed anatomic analysis of the eye area in the captive South African Painted Dogs females showed the similarities (especially in the histological examination of the eyetunics and orbital glands) as well as the differences between the Painted dog and the other representatives of *Canidae*. The differences included the shape and size od the orbita with comparison to the domestic dog. Such differences in the orbit measurements are most likely associated with the skull type, which are defined in relation to domestic dogs. The presented results significantly expand the existing knowledge on comparative anatomy in the orbit, eye and chosen accessory organs in wild *Canidae*.

## Introduction

The South African Painted Dogs (*Lycaon pictus*) belongs to the *Canidae* family of the *Caniformia* suborder and the *Carnivora* order and naturally occurs only in Africa, where it belongs to a relic line of dogs [1]. It is the only representative of the *Lycaon* genus and has a very strong herd instinct causing it to live and hunt in packs. It is commonly called the “painted dog” due to its appearance. Each individual has a unique pattern on its fur, enabling its identification [1, 2]. The South African Painted Dogs inhabits savannas and grasslands in the south-Saharan region. It is an obligate carnivore and sometimes ingests plants [3–5].

According to Smithers [6] cited by Złamał [7] the South African Painted Dogs was relatively populous and widely distributed in Africa in the 1960s. Within the last 40 years this species has almost completely disappeared from West and Central Africa, while it inhabits sparsely populated areas of East and South Africa [7, 8]. The IUCN Red List of Threatened Species [9] reports that the total population of the *Lycaon pictus* is estimated at 39 subpopulations containing 6.600 adults, of which 1.400 are reproductive. *Lycaon pictus* has predominantly diurnal habits [10]. Consequently, the isolation of subpopulations of this species leads to inbreeding and an increased risk of developing infectious diseases transmitted by domestic dogs (rabies, distemper and coronavirus diseases), decreasing its population [1, 11, 12].

There are currently 575 individuals kept in 106 zoos worldwide (328 males, 231 females and 16 individuals of undetermined sex). Of those animals, 224 are kept in 41 zoos in Europe (128 males and 96 females), whereby three females are kept in one zoo in Poland (data from 18 December 2019) [13].

Access to data on ophthalmic parameters in individual animal species is essential for the diagnosis of ophthalmologic diseases [14]. To date, the anatomic structure of the orbit, eyeball and accessory organs of the eye at a macroscopic and microscopic level have not been described in the South African Painted Dogs. There are no clinical case reports of the disease of these organs in this species. Due to the close relationship of the South African Painted Dogs to canines from the *Canis*), *Cuon*, *Cerdocyon*, *Atelocynus*, *Dusicyon*) and *Lycalopex* genera it may be assumed that they suffer from similar orbital diseases to those described in the domestic dog (*Canis lupus familiaris*).

The aim of this study was to perform an analysis of chosen structures in the examined mammals living under natural conditions. The conducted study complements the current knowledge of the comparative ocular anatomy in domesticated and wild animals. Moreover, this study provides data that may be used to develop techniques and treatment methods for diseases of the eyeball, eyelids and eyelid glands in this species by veterinarians in zoos and national parks. Due to the unique nature of the study, the results may provide useful information for future comparative studies performed by veterinary specialists in zoos. This, in turn, may improve the medical care delivered to the *Lycaon pictus*.

## Materials and methods

### Animals

**Fig 1:**
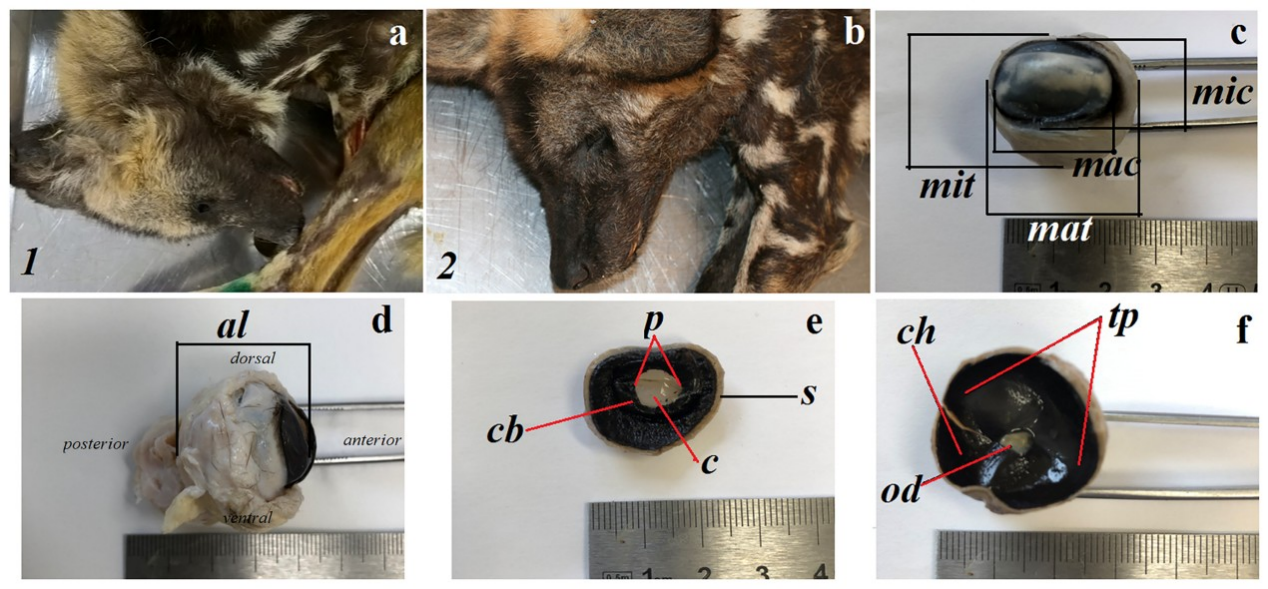
The macrograph of the female South African painted dogs and dimension of the eyeball and anatomy of the eye tunics. (a) 1—first female (13 years and 9 months old). (b) 2—second female (7 years and 1 month old). (c) anterior view of the eyeball. (d) lateral view of the eyeball (see *dorsal*, *ventral*, *anterior* and *posterior* orientation of the eyeball). (e-f) internal view of the eyeball surface. *al*—axial, *c*—cornea, *cb*—ciliary body, *ch*—choroid, *mac*—maximum corneal, *mat*—maximum transverse, *mic*—minimum corneal, *mit*—minimum transverse, *od*—optic disc, *p*—pupil, *s*—sclera, *tp*—*tapetum lucidum*.

The study was performed on two adult female (13 years 9 months old and 7 years 1 month old) captive South African Painted Dogs (*Lycaon picus pictus*) from the Wroclaw Zoological Garden (Poland) (Fig 1a and 1b). The *Lycaon pictus pictus* is in the *Lycaon* genus of the *Fissipedia* superfamily of the *Caniformia* suborder and the *Carnivora* order [9, 15]. According to the IUCN Red List of Threatened Species [9] the African wild dog is endangered (EN). The research material was collected in 2017 (first female) and 2019 (second female). The animals were not killed for the purpose of this study and were obtained *post-mortem*.

### Ethical statement

Registered persmissions for the post-mortem collection of specimens was issued by the District Veterinary Officer in Wroclaw (Poland) (No. PIW Wroc. UT-45/5/16, No. PIW Wroc. UT- 45/6/16, No. PIW Wroc. UT-45/8/16). According to the Polish and European law, studies on tissues obtained *post-mortem* do not require an approval of the Ethics Committee (Directive of the European Parliament 2010/63/UE from 22 September 2010 on the protection of animals used for scientific purposes and the Journal of Laws of the Republic of Poland from 15 January 2015 on the protection of animals used for scientific and educational purposes).

### Anatomical dissection

Eyeballs, the upper and lower eyelids, the superficial gland of the third eyelid, the third eyelid and the lacrimal gland were obtained bilaterally from both female South African Painted Dogs. The muscles of the eyeball, the orbital fat body and the fascial sheath of the eyeball were removed for histological and histochemical studies.

### Morphometry

**Fig 2:**
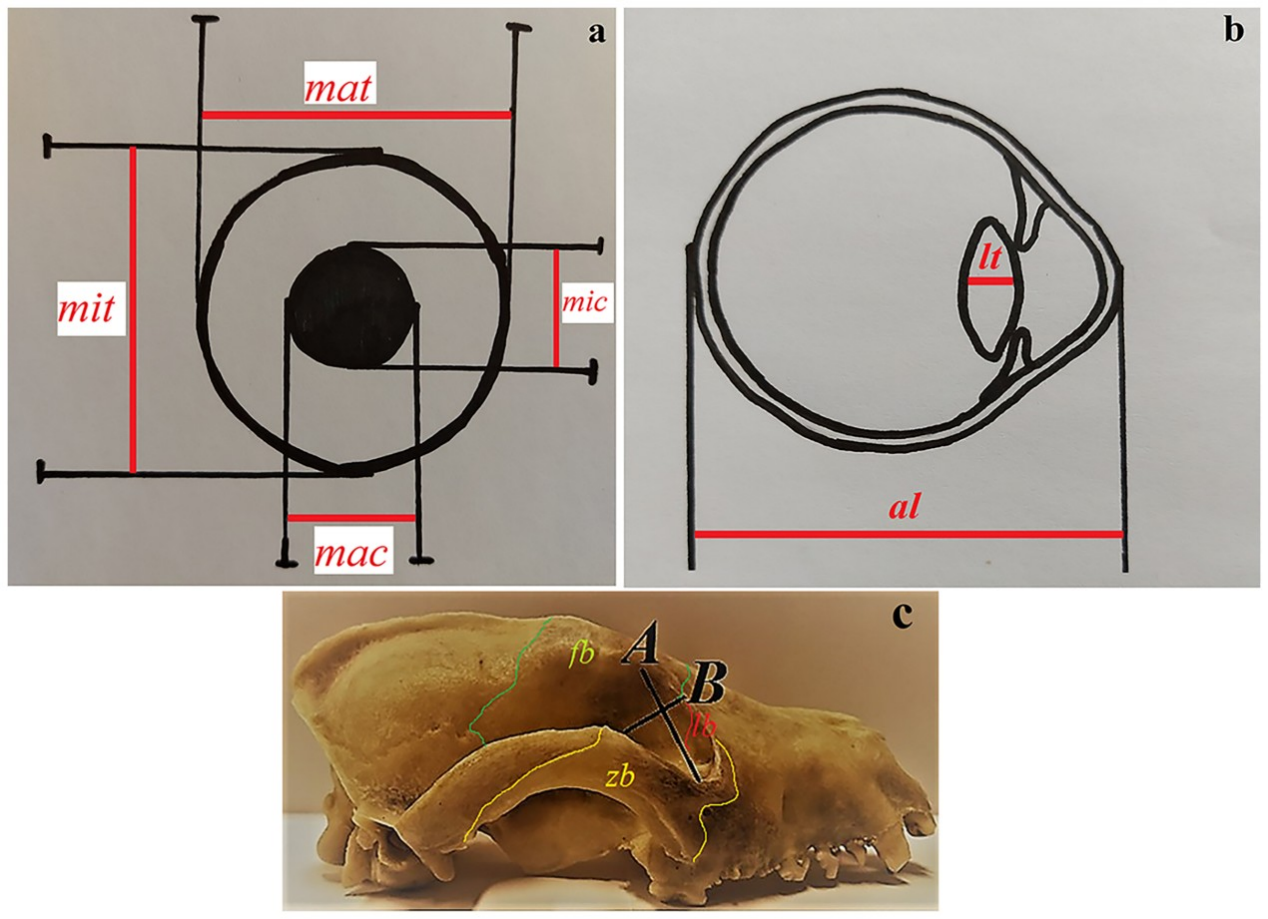
Schematic draw of the eyeball measurements and orbital parameters of the female South African painted dogs. (a) rostral view of the eyeball measurements. (b) lateral view of the eyball measurements. (c) lateral view of the skull with orbital parameters. *al*—axial length, *fb*—frontal bone, *lb*—lacrimal bone, *lt*—lens thickness, *mac*—maximum corneal, *mat*—maximum transverse, *mic*—minimum corneal, *mit*—minimum transverse, *zb*—zygomatic bone.

The eyeballs were measured according to the methods described by Cummings *et al*. [16], Lluch *et al*. [17], Kirk [18, 19] (Figs 1c, 1d, 2a and 2b):

A. Axial eye diameter: from the anterior cornea to the root of the optic nerve.
B. Maximum transverse (equatorial) eye diameter.
C. Minimum transverse (equatorial) eye diameter.
D. Maximum and minimum corneal diameter.
E. Corneal thickness.
F. Lens axial length.
G. Aqueous chamber depth.
H. Vitreous chamber depth.

The length, width and thickness of the eyelids and orbital glands in both females were measured using a digital caliper (Stainless Hardened, Farnell, Poland).

Due to the fact that samples from two individuals were collected, no statistical analysis was performed. The values of six measurements of the whole orbit, eyeball, eyelids and orbital glands from both females from both sides were recorded. The results were then formulated statistically into mean, standard deviation (S.D.) and coefficient of variation (CV%).

### Bony orbit anatomy

The anatomy of both orbitae was examined in the skulls of the two adult captive females South African Painted Dogs (S1 Fig). The following orbital parameters were measured based on the anatomical description of the orbit of the domestic dog [20] and crab-eating fox (*Cerdocyon thous*) [21, 22] using a measuring scale and a digital caliper (Stainless Hardened, Farnell, Poland) (Fig 2c):

A. Orbital vertical length (mm): the perpendicular distance between the supraorbital and infraorbital margins of the orbit.
B. Orbital horizontal width (mm): the distance between the rostral and caudal margins of the orbital rim.
C. Orbital index (%):
  $$\frac{\text{orbital}\;\text{width}\;(\text{B})}{\text{orbital}\;\text{length}\;(\text{A})}\;\text{x}\;100\%$$
D. Orbital depth (mm): distance between the optic foramen and center of the orbital rim.
E. Orbital area (mm^2^): 22/7 x (½ A x ½ B).
F. Interorbital distance (mm):
  F1. At the rostral level: distance between the junction of the frontolacrimal sutures on either side at the rostral margin of the orbit.
  F2. At the middle level: distance between the supraorbital borders of the orbit on either side.
  F3. At the caudal level: distance between the junctions of the zygomatic bone at the caudal margin of the orbit on either side.
G. Frontal length (mm): distance from the tip of the zygomatic process of the frontal bone to the frontolacrimal sutures.
H. Lacrimal length (mm): distance from the frontolacrimal sutures to the junction between the lacrimal and zygomatic bones.
I. Malar length (mm): distance from the junction between the lacrimal and zygomatic bones to the tip of the frontal process of the zygomatic bones.

### Histological examination

All the analysed organs were dissected directly post-mortem, measured (see *Morphometry*), then placed in 4% buffered formaldehyde for at least 72 hours and then rinsed in running water for 24 hours. They were then processed in a vacuum tissue processor—ETP (RVG3, Intelsint, Italy) and embedded in paraffin. The specimens were cut using a Slide 2003 (Pfm A.g., Germany) sliding microtome into 4 μm sections. The Azan trichrome, hematoxylin and eosin, Masson-Goldner trichrome, Movat pentachrome (modified Russell Movat) and picro-Mallory trichrome staining methods were applied. Next, the slides were assessed using the Zeiss Axio Scope A1 light microscope (Carl Zeiss, Jena, Germany) and a scoring system based on a standard previously described protocol [23–26]. The histological measurements of the eye tunics were performed with the AxioVision Rel. 4.8. Software—Carl Zeiss. The values of twelve measurements of the eye tunics from one female (n = 1) was recorded. The results were then formulated statistically into mean, S.D., CV% and range.

### Histomorphometric examination of the orbital gland structure

The histological measurements of the chosen elements were carried out based on a technique described by El-Fadaly *et al*. [27]. The capsule thickness was measured in 60 randomly histological field from one animal at a 50x magnification (animal/glands). The interlobar septal thickenss was measured in 60 randomly histological field from the one animal under a 50x magnification (animal/glands). The acinar and tubule outer diameters were measured in the central fields at a 400x magnification. Measurements were taken at the widest outer diameter of the transversely cut acini and tubules from 60 randomly histological field (15 acini/animal/gland and 15 tubules/animal/gland). The histological measurements of the orbital gland structure were performed with the AxioVision Rel. 4.8. Software—Carl Zeiss.

### Histochemical examination

The histochemical evaluation of the examined structure was performed according to Spicer and Henson [28], where (–) indicated a negative reaction; (–/+) and (+) a weak reaction; (++) a moderate reaction and (+++) a strong reaction. The periodic acid-Schiff (P.A.S), the alcian blue pH 1.0 (AB pH 1.0), the alcian blue pH 2.5 (AB pH 2.5), the alcian blue pH 2.5 P.A.S (AB pH 2.5/P.A.S) and the Hale’s dialysed iron (HDI) stains were obtained [29–32].

Nomenclature from the *Nomina Anatomica Veterinaria* [33] and *Nomina Histologica Veterinaria* [34] was used to describe the studied structures.

## Results

### The eyeball morphometry and eye tunics

The eyeball in the females adults South African Painted Dogs had a spherical shape. The dimensions of the eyeballs with regard to the side of the body are presented in Fig 1a and 1b and Table 1. The morphometric analysis of the eyeball revealed that the depth in the vitreous chamber of the eyeball was greater in the right eye than in the left eye (in both females). The remaining parameters were comparable.

**Table 1:** Dimensions (mm) of the eyeball parameters of the females South African painted dog (n = 2).

Parameters	Side	Mean	S.D.	CV%	Range	Overall			
						Mean	S.D.	CV%	Range
**axial eye -diameter**	**left eyeball**	25.24	0.36	1.42	24.85–25.68	25.811	1.03	3.99	24.68–27.68
	**right eyeball**	26.376	1.32	5.01	24.68–27.68				
**maximum transverse eye—diameter**	**left eyeball**	25.635	0.58	2.26	24.98–26.58	25.830	0.48	1.85	24.98–26.58
	**right eyeball**	26.026	0.36	1.38	25.54–26.47				
**minimum transverse eye -diameter**	**left eyeball**	22.505	0.56	2.48	21.67–23.01	22.554	0.44	1.95	21.67–23.07
	**right eyeball**	22.603	0.32	1.41	22.15–23.07				
**maximum corneal—diameter**	**left eyeball**	18.77	0.29	1.54	18.32–19.02	18.875	0.34	1.8	18.32–19.57
	**right eyeball**	18.981	0.41	2.16	18.57–19.57				
**minimum corneal—diameter**	**left eyeball**	14.098	0.14	0.99	13.87–14.25	14.556	0.5	3.43	13.87–15.04
	**right eyeball**	15.015	0.12	0.79	14.87–15.04				
**corneal -thickness**	**left eyeball**	1.61	0.11	7.01	1.48–1.78	1.589	0.09	5.66	1.45–1.78
	**right eyeball**	1.568	0.08	5.1	1.45–1.68				
**aqueous chambers—depth**	**left eyeball**	5.273	0.16	3.03	7.58–8.16	5.361	0.2	3.73	7.58–8.59
	**right eyeball**	5.448	0.22	4.03	7.69–8.59				
**lens—axial length**	**left eyeball**	7.953	0.23	2.89	7.58–8.16	8.086	0.27	3.33	7.58–8.59
	**right eyeball**	8.22	0.33	4.01	7.69–8.59				
**vitreous chamber—depth**	**left eyeball**	11.233	0.32	2.48	10.68–11.97	12.201	1.51	12.37	10.68–15.98
	**right eyeball**	13.17	1.64	12.45	11.57–15.98				
***tapetum lucidum*–length**	**left eyeball**	18.375	0.32	1.74	17.99–18.78	18.578	0.68	3.66	17.64–19.87
	**right eyeball**	18.781	0.95	5.05	17.64–19.87				
***tapetum lucidum*–thickness**	**left eyeball**	11.805	0.6	5.08	11.16–12.66	11.993	0.52	4.33	11.16–13.04
	**right eyeball**	12.181	0.54	4.43	11.36–13.04				

CV%—coefficient of variation; S.D.—standard deviation

The South African Painted Dogs eyeballs consist of a fibrous layer (sclera and cornea), a vascular layer (choroid, ciliary body, iris) and an inner layer (retina) as well as a lens, an anterior, a posterior and vitreous chambers of the eyeball (Fig 1e and 1f). Histological measurements of the eyeball are presented in Table 2.

**Table 2:** Dimensions (μm) of the histological parameters of the eye tunics of female South African painted dog (n = 1).

Parameters	Mean	S.D.	CV%	Range
Sclera (equator)—thickness	437.675	13.93	3.18	419.08–453.69
Anterior corneal epithelium—thickness	53.033	4.77	8.99	49.25–60.84
Bowman’s membrane—thickness	3.893	0.45	11.55	2.91–5.11
Proper substance of cornea—thickness	1384.403	99.89	7.21	1204.4–1501.02
Descemet’s membrane—thickness	20.171	0.71	3.51	19.17–21.48
*Tapetum lucidum*—thickness	157.011	25.66	16.34	124.87–180.26
Bruch’s membrane—thickness	25.555	2.7	10.56	24.13–30.34
Pars plana of the ciliary body—thickness	64.326	6.41	9.96	56.79–73.77
Pars plicata of the ciliary body—thickness	300.853	61.32	20.38	206.45–351.55
Iris—thickness	292.856	84.99	29.02	165.29–418.61
Retina—thickness	276.168	32.14	11.63	237.56–318.26
Lens capsule—thickness	29.175	1.87	6.41	26.81–32.1

CV%—coefficient of variation; S.D.—standard deviation

**Fig 3:**
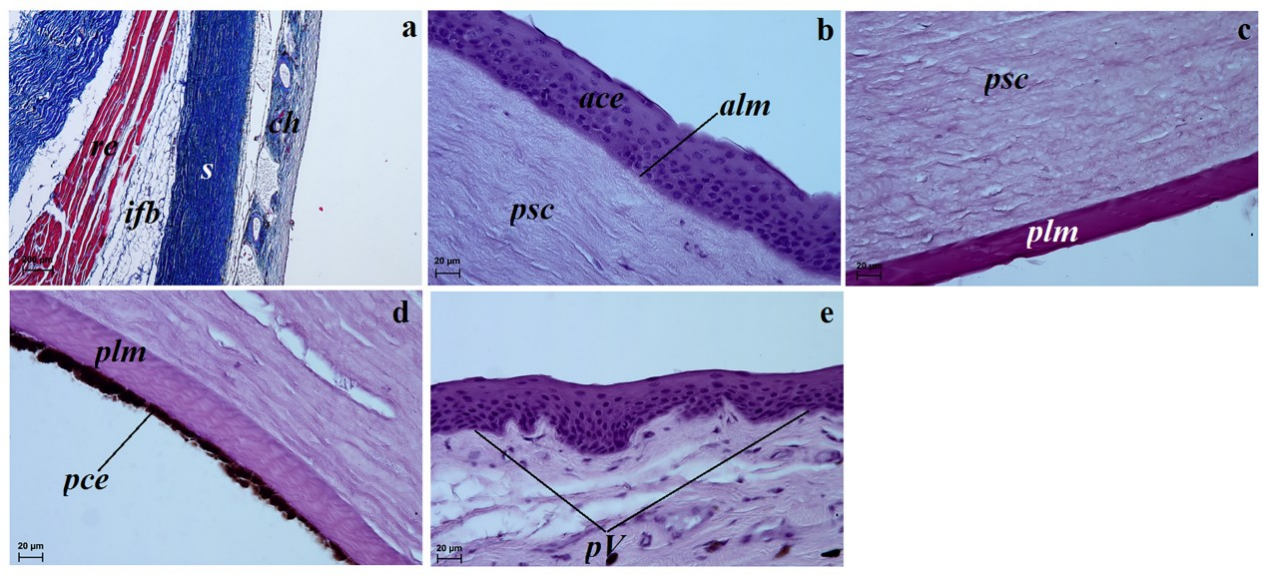
Characterization of the structure of the fibrous layer of the eyeball in the female South African painted dogs using light microscopy. (a) Sclera with the visible choroid, intraorbital fat body and retractor bulbi muscle. Azan trichrome staining. (b) The anterior corneal epithelium and anterior limiting membrane (Bowman’s membrane) and proper substance of the cornea. H&E staining. (c) The proper substance of the cornea with the posterior limiting membrane (Descemet’s membrane) with a strongly positive P.A.S. reaction. (d) The posterior corneal epithelium with numerous melanocyte aggregates. H&E staining. (e) The palisades of Vogt present in the corneal limbus epithelium. H&E staining. *ace*—anterior corneal epithelium, *alm*–anterior limiting membrane, *ch*–choroid, *s*–sclera, *ifb*–intraperiorbital fat body, *pce*–posterior corneal epithelium, *plm*–posterior limiting membrane, *psc*–proper substance of cornea, *pV*–palisades of Vogt, *re*–retractor of eyeball, Scale bars: (a) = 200 μm, (b–e) = 20 μm.

The sclera consisted of three layers: the episcleral lamina—composed of loose fibrous connective tissue; the proper substance of the sclera—consisted of collagen fibres arranged toward the surface of the eyeball, a network of elastic fibres and—the dark lamina of the sclera composed of a few fibroblasts, numerous blood vessels and pigmented cells containing melanin (Fig 3a). The external surface of the episcleral lamina was covered by a simple cuboidal epithelium.

The cornea consisted of five layers consisting of the following layers from external to internal: the anterior corneal epithelium, the anterior limiting membrane also known as Bowman’s membrane, the proper substance of the cornea, the posterior limiting membrane also known Descemet’s membrane and the posterior corneal epithelium. The anterior corneal epithelium was a non-keratinised stratified squamous epithelium consisting of from nine to twelve rows of cells (Fig 3b). The anterior limiting membrane consisted of collagen fibres. The proper substance of the cornea was the thickest corneal layer and contained fibrous connective tissue made up of dominant collagen fibers with a layered parallel structure and fewer flattened korneocytes arranged between the fiber layers (Fig 3b and 3c). The posterior limiting membrane consisted of regularly arranged collagen fibers and featured a strongly positive P.A.S`reaction (Fig 3c). Descemet’s membrane was covered by double layers of a posterior corneal epithelium containing numerous large melanocyte aggregates providing the characteristic brown colour of this membrane in the females (Fig 3d).

The corneal limbus was located at the border between the sclera and the cornea. Palisades of Vogt were present within the epithelium of the the corneal limbus (Fig 3e). In the examined females, the limbal epithelium was composed of 9 layers of epithelial cells: 2 layers of flattened superficial squamous cells, 5 layers of intermediate wing cells and 2 layers of basal cells.

**Fig 4:**
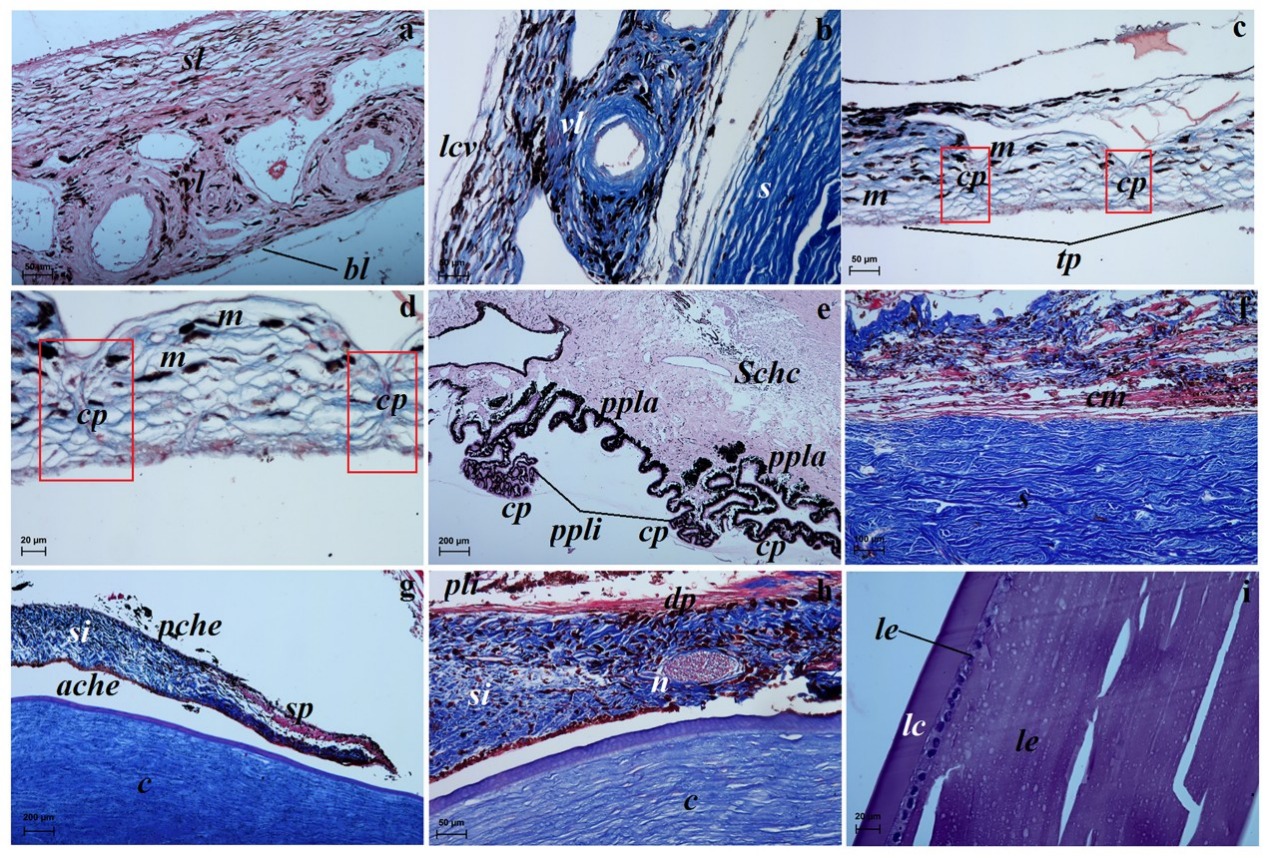
Characterisation of the structure of the vascular layer and lens of the eyeball in the females South African painted dog using light microscopy. (a, b) The choroid layers. Movat pentachrome staining (a) and Masson trichrome staining (b). (c, d) The cellular *tapetum lucidum*. picro-Mallory trichrome staining. (e) The ciliary body with visible pars plana and pars plicata and ciliary processes. H&E staining. (f) The ciliary body with ciliary muscle. picro-Mallory trichrome staining. (g) The iris with of the sphincter pupil. picro-Mallory trichrome staining. (h) The iris with of the dilator pupil. picro-Mallory trichrome staining. (i) The lens with visible lens capsule and lens epithelium. H&E staining. *ache*–anterior chamber of eyeball, *bl*—basal lamine, *c*–cornea, *cp* in red square—capillary, *cm*–ciliary muscle, *cp*–ciliary processes, *dp*–dilator of pupil, *lc*–lens capsule, *lcv*–lamina of capillary vessels, *le*–lens epithelium, *lf*–lens fibres *m*–melanocytes, *pche*–posterior chamber of eyeball, *pli*–pigmented layer of iris, *ppla*–pars plana, *ppli*—pars plicata, *s*–sclera, *Schc*—Schlemm’s canal, *si*–stroma of iris, *sl*–suprachoroid layer, *sp*–sphincter of pupil, *tp*–*tapetum lucidum*, *vl*—vascular layer. Scale bars: (a–c, h) = 50 μm, (d, i) = 20 μm, (e, g) = 200 μm, (f) = 100 μm.

The choroid in the examined South African Painted Dogs consisted of five layers: the suprachoroid layer—composed of fibrous connective tissue, forming lamellae separated from one another by slit-shaped spaces. This layer was characterised by a large number of melanocytes. The second layer—the vascular layer—was characterised by ciliary arteries and large diameter vorticose veins. Loose connective tissue with collagen and elastin fibers and melanocytes were present between the blood vessels (Fig 4a and 4b). The third layer—the cellular *tapetum lucidum*—was composed of oval elongated cells in the center, progressively thinning and eventually disappearing towards the periphery (Fig 4c and 4d). This tapetum was composed of multiple layers of cells (maximum 14–17 layers of tapetal cells) arranged in a bricklike structure (Fig 4c and 4d). In the studied samples, numerous melanocyte aggregates were observed between the layers of tapetal cells (Fig 4d). The *tapetum lucidum* was thick in its central part and its thickest portion was located dorsally to the optic nerve disc. The *tapetum lucidum* was semi-circular and milky (Fig 1f). The peripheral zone of this *tapetum lucidum* was covered by the lightly pigmented retinal epithelial layer. The fourth layer—the lamina of the capillary vessels—was composed of fenestrated vessels and absent melanocytes. The fifth layer was the basal layer also known as Bruch’s membrane and consisted of a capillary basal membrane, collagen and elastic fibers (Fig 4a and 4b).

The ciliary body was big and occupied a relatively wide zone (Fig 1e). It was round and lined with a double layer of a cuboid epithelium. The deeper epithelial layer was rich in pigment and formed an extension of the epithelial layer of the retina. The superficial layer of the epithelium lacked pigment and formed an extension of the receptor layer of the retina. The pars plana of the epithelium was markedly irregular and folded (Fig 4e). The pars plicata formed a ruffled portion of the ciliary body (Fig 4e). It also had numerous radial ridges that formed the ciliary processes at the posterior chamber of the eyeball (Fig 4e). The ciliary body contained a strongly developed ciliary muscle (Fig 4f).

The iris was brown. It was composed of the anterior iris epithelium, which was a simple squamous epithelium; the outer limiting layer was composed of fibrocytes and collagen fibres; the stroma of the iris consisted of numerous pigment cells, fibrocytes, blood vessels, collagen fibres, nerves and two smooth muscles: the sphincter muscle and the dilatator muscle (Fig 4g and 4h); and the posterior surface of the retina forming an extension of the retinal pigment layer (Fig 4h). The pupil in the examined animals was round (Fig 1e).

Similarly to other mammals, the retina contained a pigmented area (1 layer) and a visual area (9 layers). The pigmented area contained a simple cuboidal epithelium, whose cells contained melanin granules (Fig 4h).

The shape of lens in the females South African Painted Dogs was biconvex round. The anterior surface contained a simple lens epithelium, which was absent on the posterior surface (Fig 4i). The whole lens was surrounded by an accelular lens capsule (Fig 4i). The mass of the lens was mostly made up of lens fibers.

### Bony orbit anatomy

**Fig 5:**
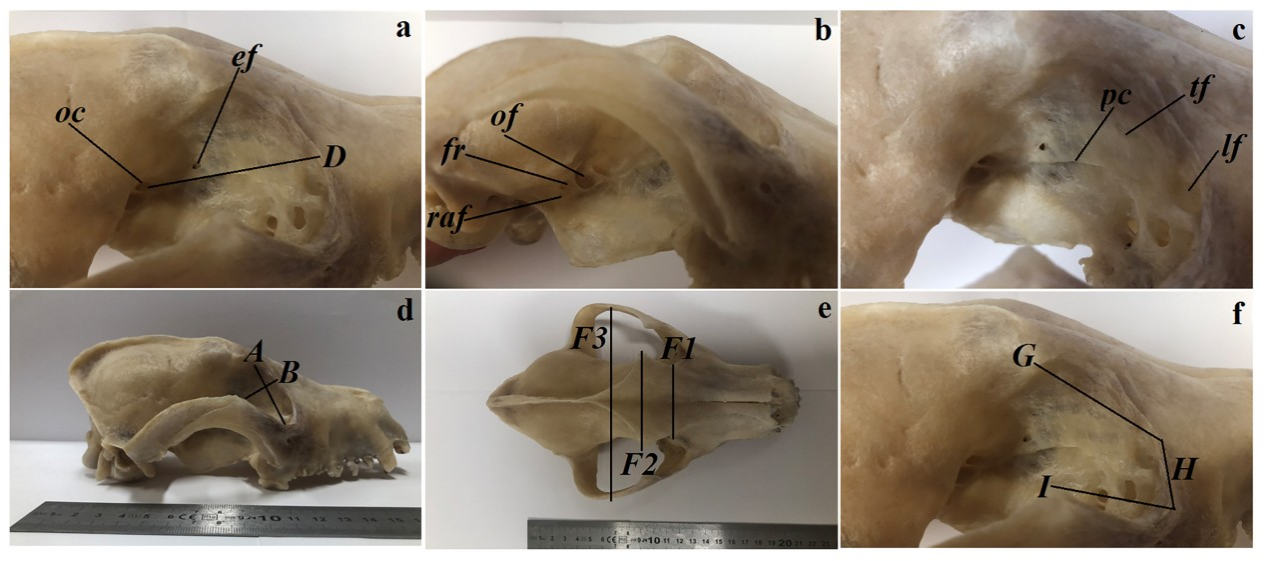
The macrograph of the bony orbit anatomy and orbital parameters of the females South African painted dog. (a-d,f) lateral view of the orbita. (e) dorsal view of the skull. *ef*–ethmoidal foramen, *fr*–foramen rotundum, *lf*–lacrimal foramen, *oc*—optic canal, *of*–orbital fissure, *pc*–pterygoid crest, *raf*–rostral alar foramen, *tf*–trochlear fovea, *A*–orbital vertical length, *B*–orbital horizontal width, *D*–orbital depth, *F1*—at rostral level, *F2*—at middle level, *F3*—at caudal level, *G*–frontal length, *H*–lacrimal length, *I*–malar length.

The orbit in the two female South African Painted Dogs was funnel (conical) shaped (Fig 5a) and was open. The orbital ring complemented the orbital ligament connecting the zygomatic process of the frontal bone with the frontal process of the zygomatic bone. The length of this ligament is presented in Table 3.

**Table 3:** Dimensions of the orbital parameters of the skull of the females South African painted dog (n = 2).

Parameters	Side	Mean	S.D.	CV%	Range	Overall			
						Mean	S.D.	CV%	Range
**Orbital vertical length (mm)**	**left orbit**	35.783	0.68	1.9	34.87–36.68	36.13	0.64	1.77	34.87–36.98
	**right orbit**	36.477	0.37	1.01	35.98–36.98				
**Orbital horizontal width (mm)**	**left orbit**	33.1	0.59	1.78	32.18–34.01	32.875	0.52	1.58	32.18–33.14
	**right orbit**	32.65	0.31	0.94	31.87–33.14				
**Orbital index (%)**	**left orbit**	92.507	1.37	1.48	90.34–94.92	91.021	2.08	2.28	90.34–91.99
	**right orbit**	89.535	1.37	1.53	88.06–91.99				
**Orbital depth (mm)**	**left orbit**	51.392	1.56	3.03	49.4–53.42	51.342	1.19	2.31	49.4–52.13
	**right orbit**	51.292	0.61	1.18	50.47–52.13				
**Orbital area (mm**^**2**^**)**	**left orbit**	930.718	31.73	3.4	894.55–976.42	933.139	23.458	2.51	894.55–946.9
	**right orbit**	935.56	12.61	1.34	900.68–946.9				
**Interorbital distance (mm):**	**at rostral level**	41.428	0.64	1.54	40.65–42.36	n. a	n. a	n. a	n. a
	**at middle level**	53.37	0.84	1.49	51.96–54.96	n. a	n. a	n. a	n. a
	**at caudal level**	93.572	0.82	0.87	92.35–94.9	n. a	n. a	n. a	n. a
**Frontal length (mm)**	**left orbit**	32.414	0.51	1.57	31.58–33.58	32.152	1.07	3.32	31.58–33.46
	**right orbit**	31.89	1.42	4.45	29.78–33.46				
**Lacrimal length (mm)**	**left orbit**	24.265	0.59	2.43	23.09–25.07	23.047	1.51	6.55	23.09–23.25
	**right orbit**	21.829	0.93	4.26	20.34–23.25				
**Malar length (mm)**	**left orbit**	28.175	0.45	1.59	27.66–29.11	30.031	1.98	6.59	27.66–33.06
	**right orbit**	31.886	0.84	2.63	30.63–33.06				
**Orbital ligament length (mm)**	**left orbit**	28.172	0.37	1.31	27.64–28.68	28.381	0.72	2.53	27.48–29.79
	**right orbit**	28.591	0.94	3.28	27.48–29.76				

CV%—coefficient of variation; n. a—not applicable; S.D.—standard deviation.

There was a single ethmoid opening for the ethmoid nerve on the orbital lamina (Fig 5a). The following openings were found in the sphenoid bone beginning cranially: the optic canal for the optic nerve; the orbital fissure for the external ocular vein, optic, oculomotor, trochlear and abducens nerves; the foramen rotundum for the maxillary nerve and the rostral alar foramen for the maxillary artery (Fig 5a and 5b). Behind the line formed by these openings lay the prominent crista pterygoidea (Fig 5c). Anterior and beneath the zygomatic arch of the frontal bone lay a slight indentation—the trochlear notch for the dorsal oblique muscle of the eye (Fig 5c). A large and wide lacrimal foramen was found on the orbital surface of the lacrimal bone, just behind the orbital rim (Fig 5c). The fossa of the lacrimal sac and the groove for the ventral oblique muscle were not detected in the studied females.

The orbit dimensions of the studied animals taking into account sides have been presented in Table 3 and Fig 5d–5f. The orbital vertical length was found to be larger in the right orbit, while the orbital horizontal width was larger in the left orbit. The orbital area was larger in the right orbit, while the lacrimal length was greater in the left orbit.

### The upper and lower eyelids

#### Gross anatomy and morphometrics

**Fig 6:**
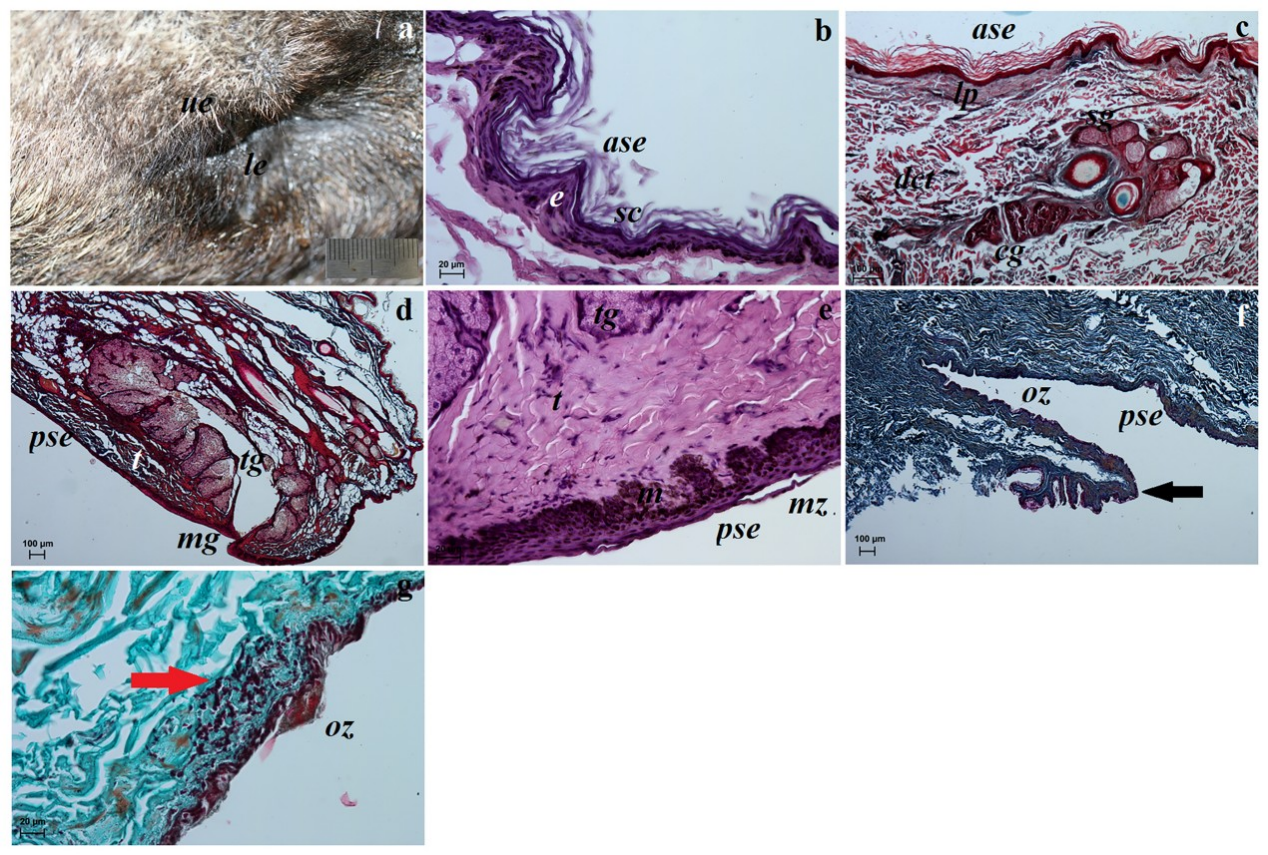
The macrograph (a) and histological images (b—g) of the upper and lower eyelids of the females South African Painted Dog. (b, e)—H&E staining, (c, d)—Movat pentachrome staining, (f)—picro-Mallory trichrome staining, (g)—Masson-Goldner trichrome. *ase*—anterior surface of eyelid, *black arrow*—conjunctival fold, *cg*—ciliary glands, *dct*—dense connective tissue, *e*—epithelium, *lp*—lamina propria, *m*—melanocytes, *mg*—marginal zone, *oz*—orbital zone, *red arrow*—lymphoid follicle, *sc*—stratum corneum, *sg*—sebaceous glands, *t*—tarsus, *tg*—tarsal gland. Scale bars: (a) = 2 cm, (b, e, g) = 20 μm, (c, d, f) = 100 μm.

The upper eyelid and lower eyelid in the captive adult South African Painted Dogs appeared to correspond to the eyelid macromorphology of the standard domestic dogs [35]. In the African wild dog, 2–3 rows of eyelashes were present on the anterior palpebral margin of the upper eyelid. They were absent in the lower eyelids. The palpebral conjunctiva was strongly pigmented and brown. The upper eyelid of the examined females was more mobile and larger than the lower eyelid (Fig 6a). The morphometric parameters of the upper and lower eyelids (length, width, thickness) in the two female South African Painted Dogs are presented in Table 4. The obtained parameters were comparable between the two females.

**Table 4:** Dimensions (mm) of the macroscopic parameters of eyelids and orbital glands of the females South African painted dog (n = 2).

Parameters	Side	Mean	S.D.	CV%	Range	Overall			
						Mean	S.D.	CV%	Range
**upper and lower eyelid—length**	**left**	26.135	0.35	1.33	25.6–26.99	26.791	0.86	3.21	25.6–28.64
	**right**	27.448	0.78	2.84	26.69–28.64				
**upper eyelid—width**	**left**	13.675	0.32	3.34	13.23–14.22	13.885	0.38	2.73	13.23–15.01
	**right**	14.095	0.36	2.55	13.58–15.01				
**upper eyelid—thickness**	**left**	5.13	0.05	0.97	5.07–5.21	5.205	0.18	3.45	4.99–5.55
	**right**	5.28	0.26	4.92	4.99–5.55				
**lower eyelid—width**	**left**	10.011	0.27	2.69	9.6–10.36	10.078	0.41	4.06	9.6–11.2
	**right**	10.145	0.58	5.71	9.85–11.2				
**lower eyelid—thickness**	**left**	5.09	0.13	2.55	4.87–5.36	5.617	0.55	9.79	4.87–6.38
	**right**	6.145	0.18	2.92	5.89–6.38				
**third eyelid—length of crossbar**	**left**	15.875	0.21	1.32	15.64–16.13	16.072	0.36	2.23	15.36–16.98
	**right**	16.269	0.47	2.88	15.36–16.98				
**third eyelid—length of upper and lower branch**	**left**	21.971	0.24	1.09	21.58–22.21	22.167	0.62	2.79	21.34–23.36
	**right**	22.363	0.87	0.93	21.34–23.36				
**superficial gland of the third eyelid—length**	**left**	15.65	0.41	2.61	15.04–16.21	16.062	0.65	4.04	15.04–17.32
	**right**	16.475	0.72	4.37	15.54–17.32				
**superficial gland of the third eyelid—width**	**left**	12.623	0.38	3.01	11.98–13.25	12.621	0.44	3.48	11.35–13.33
	**right**	12.62	0.41	3.24	11.35–13.33				
**superficial gland of the third eyelid—thickness**	**left**	3.828	0.23	6.01	3.54–4.12	3.894	0.24	6.16	3.54–4.21
	**right**	3.96	0.25	6.31	3.57–4.21				
**lacrimal gland—length**	**left**	18.81	0.31	1.64	18.24–19.31	18.652	0.65	3.48	17.22–19.36
	**right**	18.495	0.9	4.86	17.22–19.36				
**lacrimal gland—width**	**left**	7.88	0.34	4.31	7.49–8.34	8.192	0.43	5.24	7.49–8.97
	**right**	8.505	0.43	5.05	7.96–8.97				
**lacrimal gland—thickness**	**left**	2.976	0.42	14.11	2.62–3.65	3.019	0.33	10.93	2.62–3.65
	**right**	3.061	0.28	7.75	2.65–3.62				

CV%—coefficient of variation; S.D.—standard deviation.

#### Histological and histochemical examinations

**Fig 7:**
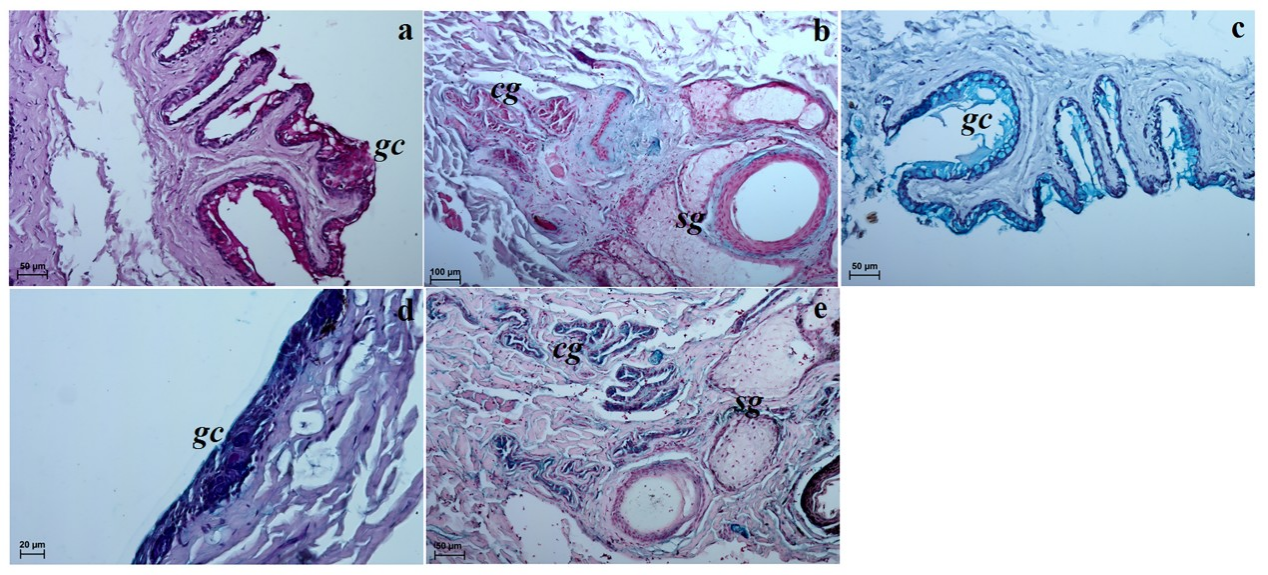
The histochemical images of the upper and lower eyelids of the females South African painted dog. (a) P.A.S. positive reaction (+++) in goblet cells located in posterior surface of eyelids. (b) AB pH 1.0 negative reaction (–) in the sebaceous and ciliary glands. (c) AB pH 2.5 negative reaction (–) in the sebaceous glands and slightly positive reaction (–/+) in the ciliary glands. (d) AB pH 2.5 P.A.S. strongly positive reaction (+++, violet color) in the goblet cells in posterior surface of eyelid. (e) HDI negative reaction (–) in the sebaceous gland and weakly positive reaction (–/+) in the ciliary glands. *cg − ciliary glands*, *gc—goblet cells*. Scale bars: (a, c, e) = 50 μm, (b) = 100 μm, (d) = 20 μm.

The upper eyelid and lower eyelids in the South African Painted Dogs contained an anterior surface covered by a stratified squamous epithelium with 9 to 13 layers of nucleated cells (Fig 6b). The superficial layer of the stratified squamous epithelium composed a thin layer of the stratum corneum (71.124 ±14.58 μm thick) (Fig 6b). Numerous melanin granules were found in the the stratum basale (Fig 6b). A thin lamina propria formed from loose connective tissue was present under the epithelium (Fig 6c). The stroma of both eyelids was formed from dense irregular connective tissue with a network of collagen and elastic fibers, large arteries, small veins, numerous nervous fibres and a thick layer of bundles of muscle. The sebaceous glands opened into the follicles of the eyelashes on the upper eyelid, while the ciliary glands were coiled, tubular and secreted into hair follicles (Fig 6c). Elongated vesicular tarsal glands (more developed in the upper eyelids) were found in both eyelids and opened into the posterior palpebral margins (Fig 6d). A histochemical analysis of the sebaceous glands, ciliary glands and tarsal glands is presented in Table 6 and Fig 7b–7e. A clearly marked superior and inferior tarsus, which consisted of dense fibrous connective tissue (Fig 6d) was also noted. The posterior surface of both eyelids consisted of two parts—the marginal zone and the orbital zone (Fig 6e and 6f). The marginal zone was covered by a stratified columnar epithelium with from five/six to seven/eight layers of cells. The orbital zone, on the other hand, was covered by five to seven non-keratinized layers of numerous goblet cells. One to two extra conjunctival folds (visible in the orbital zone) were found in the posterior surface of the upper eyelids and contained numerous goblet cells (non lymphoid regionand fewer melanocyte aggregates (Fig 6f). The goblet cells in both eyelids revealed a strongly positive histochemical reaction (Table 6, Fig 7a, 7c and 7d). The presence of a single conjunctival lymph nodule aggregate was seen in the orbital zone of the lymphoid region in the lower eyelids. That area lacked goblet cells (Fig 6g).

### The superficial gland of the third eyelid

#### Shape, location and morphometrics

**Fig 8:**
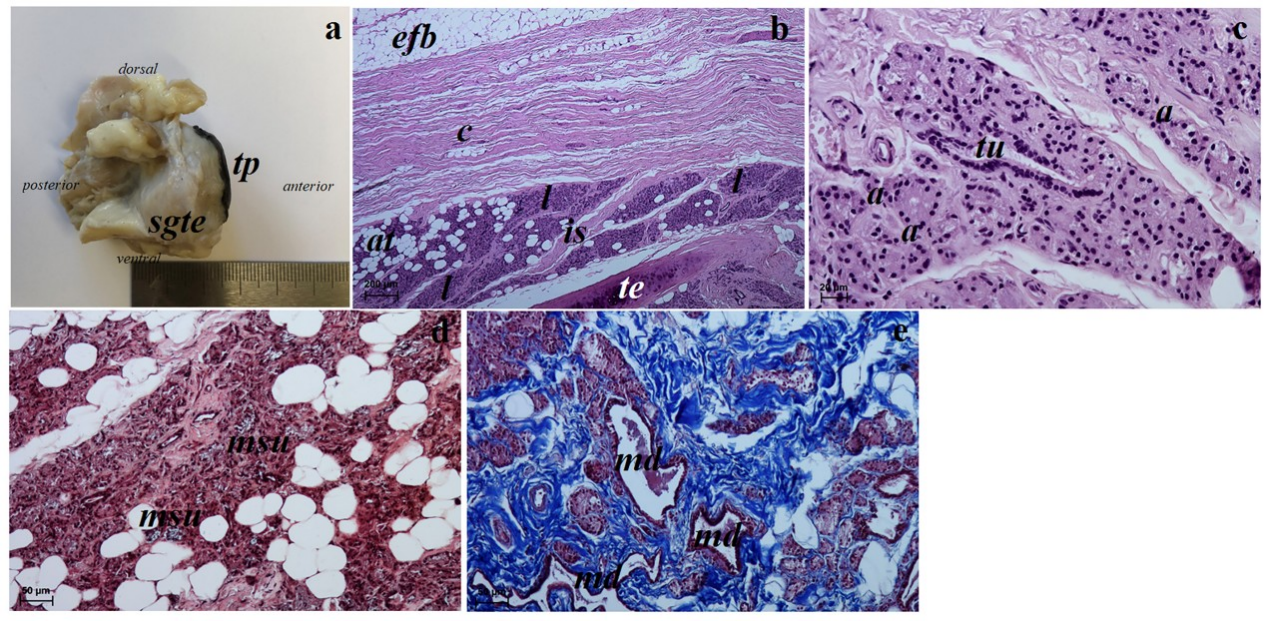
The macrograph (a) and histological images (b–e) of the superficial gland of the third eyelid of the females South African Painted Dog. (a) lateral view of the third eyelid with the superficial gland of the third eyelid (see *dorsal*, *ventral*, *anterior* and *posterior* orientation of the eyeball). (b, c) superficial gland of the third eyelid. H&E staining. (d) superficial gland of the third eyelid. Movat pentachrome staining. (e) superficial gland of the third eyelid. picro-Mallory trichrome staining. *a*–acini, *at*—adipose tissue, *c*–capsule, *efb*–extraperiorbital fat body, *is*–interlobar septa, *l*–lobes, *md*–main ducts, *msu*–mucous secretory units, *sgte*–superficial gland of the third eyelid, *tp—palpebra tertia* (third eyelid), *tu*–tubules. Scale bars: (a) = 6 cm, (b, c) = 200 μm, (d, e) = 50 μm.

The superficial gland of the third eyelid was oval and light pink (Fig 8a). It was located in the medial canthus of the eye between the medial straight and ventral straight muscles and was partially covered by the ventral oblique muscles. The morphometric parameters of the superficial gland of the third eyelid (length, width, thickness) in the two female South African Painted Dogs are presented in Table 4. The obtained results were highly compatible.

#### Histological and histochemical examinations

**Fig 9:**
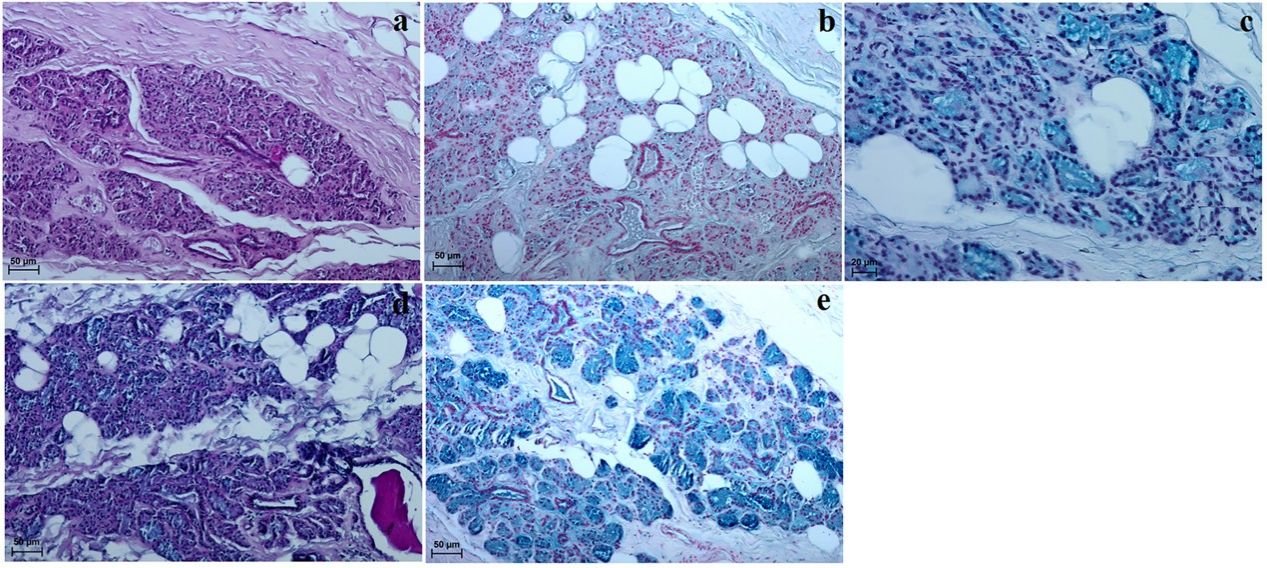
The histochemical images of the superficial gland of the third eyelid of the females South African painted dog. (a) P.A.S. weakly positive reaction (+) in the acini. (b) AB pH 1.0 slightly reaction (–/+) in the secretory cells. (c) AB pH 2.5 middle positive reaction (++) in the glandular acini. (d) AB pH 2.5 P.A.S. weakly positive reaction (+, blue color) in the acini. (e) HDI strongly positive reaction (+++) in the secretory cells. Scale bars: (a, b, d, e) = 50 μm, (c) = 20 μm.

The superficial gland of the third eyelid had a multilobar tubuloacinar structure and a serous nature (Table 6 and Fig 9). It was surrounded by a large intraperiorbital fat body, under which lay a thick connective tissue capsule, which was infiltrated with adipocytes in numerous areas (Fig 8b). Both thick and thin interlobar septae ran from the connective tissue capsule, which were also infiltrated with numerous adipocytes (Fig 8b). The interlobar septae divided this gland structure into big and small lobes (Fig 8b). The connective tissue capsule was composed of collagen and elastic fibers, arteries, veins and nerves. Each lobe consisted of acini and tubules. Numerous adipocytes were also present between the secretory units. The latter were composed of a small lumen constituting tall conical cells with an eosinophilic cytoplasm (Fig 8c). The tubules with a large lumen were composed of a single layer of cubic cells (Fig 8c). The Movat-pentachrome stain showed the presence of single faintly marked mucous secretory units (Fig 8d). Numerous main ducts were observed in the central areas of the lobes as well as within the interlobar septae, which were composed of a simple columnar epithelium (Fig 8e). Goblet cells were not found in the main ducts of either of the studied females. Histological measurements of the superficial gland of the third eyelid are presented in Table 5.

**Table 5:** Dimensions (μm) of the histological parameters of the orbital glands of females South African painted dog (n = 2).

Parameters		Mean	S.D.	CV%	Range
**Superficial gland of the third eyelid**	**capsule thickness**	474.533	66.06	13.92	401.38–557.9
	**interlobar septal thickness**	91.825	29.6	32.23	61.79–112.54
	**acini outer diameter**	36.666	4.28	11.67	32.33–42.06
	**tubular outer diameter**	77.69	17.81	22.93	61.87–106.06
**Lacrimal gland**	**capsule thickness**	236.571	131.49	55.58	144.81–469.26
	**interlobar septal thickness**	97.51	28.07	28.78	70.38–133.74
	**acini outer diameter**	34.813	8.86	24.7	24.6–47.14
	**tubular outer diameter**	81.943	32.14	39.22	56-05-123.17

CV%—coefficient of variation; S.D.—standard deviation.

### The third eyelid

#### Shape, location and morphometric

**Fig 10:**
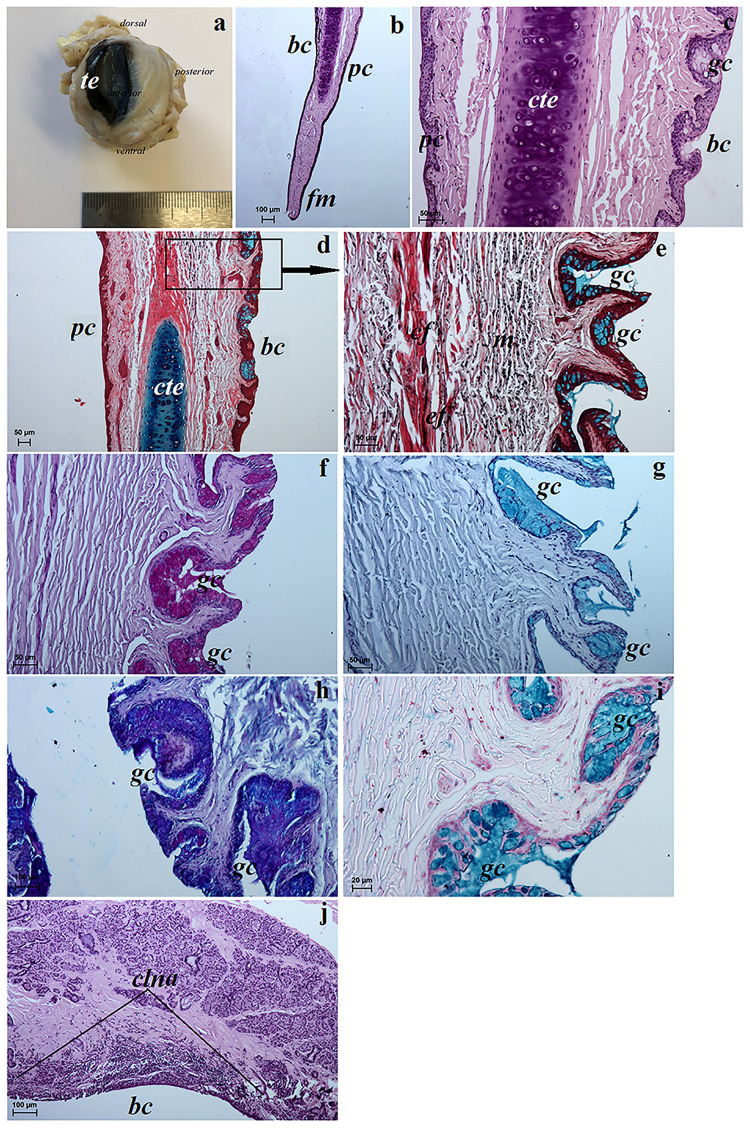
The macrograph (a) and histological (b–e, j) and histochemical images (f–i) of the third eyelid of the females South African Painted Dog. (a) view of the third eyelid. (b, c, j) histological view of the third eyelid. H&E staining. (d, e) histological view of the third eyelid. Movat pentachrome staining. (f) P.A.S. strongly positive reaction (+++) in the goblet cells located in the bulbar conjunctiva and conjunctival sac. (g) AB pH 2.5 strongly positive reaction (+++) in the goblet cells. (h) AB pH 2.5 P.A.S. middle positive reaction (++, violet color) and strongly positive reaction (+++, blue color) in the goblet cells. (i) HDI positive reaction (++/+++) in the goblet cells. *bc*–bulbar conjunctiva, *cf*—collagen fibres, *clna*–conjunctival lymph noduli aggregate, *cte*—cartilage of the third eyelid, *ef*—elastic fibres, *fm*—free margin, *gc*–goblet cells, *m*–melanocytes, *pc*—palpebral conjunctiva, *te*–third eyelid. Scale bars: (a) = 5 cm, (b, h, j) = 100 μm, (c–g) = 50 μm, (i) = 20 μm.

The third eyelid of the female South African Painted Dogs was located in the medial canthus of the eye. The macroscopically marginal part of the third eyelids was strongly pigmented and thick (Fig 10a). The third eyelid was T-shaped and composed of cartilage, which consisted of an upper and lower branch and a crossbar. This crossbar were surrounded by the superficial gland of the third eyelid. The morphometric parameters of the third eyelid (length of the crossbars and upper and lower branches combined) are presented in Table 4. The obtained results were comparable.

#### Histological and histochemical examinations

The palpebral conjunctiva of the third eyelid of the examined South African Painted Dogs was covered by a non-keratinised stratified squamous epithelium with four to thirteen layers of nucleated cells (Fig 10b and 10c). In contrast, the bulbar conjunctiuva of the third eyelid contained a stratified columnar epithelium with three to eight layers of epithelial cells (Fig 10b and 10c). The bulbar conjuctiva of the free margin of the third eyelid featured numerous melanocytes. The cartilage of the third eyelid was surrounded by a thick layer of collagen and elastic fibres (Fig 10d and 10e). The cartilage was composed of hyaline tissue with numerous chondrocytes and little intercellular substance (Fig 10c). The stroma of the third eyelid was formed from a network of dense collagen and elastic fibres and numerous melanocytów (Fig 10e). Numerous goblet cells were present in the bulbar conjunctiva and the conjunctival sac (Fig 10c–10e). These goblet cells characterized a strongly P.A.S, AB pH 2.5, AB pH 2.5 P.A.S and HDI positive reaction and weakly AB pH 1.0 positive reaction (Table 6 and Fig 10f–10i). We found two—to three areas of conjunctival lymph nodule aggregates within the bulbar conjunctiva of the third eyelid (Fig 10j).

**Table 6:** Histochemical examination of the upper and lower eyelid, superficial gland of the third eyelid, third eyelid and lacrimal gland of the South African painted dog (n = 2).

	P.A.S	AB pH 1.0	AB pH 2.5	AB pH 2.5 P.A.S	HDI
**Tarsal glands**	–	–	–	–	–
**Sebaceous glands**	–	–	–	–	–
**Ciliary glands**	–/+	–	–/+	–/+ (blue color)	–/+
**Posterior surface of the upper and lower eyelids—goblet cells**	+++	++	+++	+++ (violet color)	+++
**Superficial gland of the third eyelid—acini**	+	–/+	++	+ (blue color)	+++
**Bulbar conjunctiva of the third eyelid and conjunctival sac—goblet cells**	+++	–/+	+++	++ (violet color)	++/+++
				+++ (blue color)	
**Lacrimal gland—acini**	+++	+	+	+++ (violet color)	+/++

AB pH 1.0—the alcian blue pH 1.0; AB pH 2.5—the alcian blue pH 2.5; AB pH 2.5 P.A.S—the alcian blue pH 2.5 P.A.S; HDI—the Hale’s dialysed iron; P.A.S.—the periodic acid-Schiff; +++ a strong reaction; ++ a moderate reaction; + and -/+ a weak reaction;—a negative reaction.

### The lacrimal gland

#### Shape, location and morphometrics

**Fig 11:**
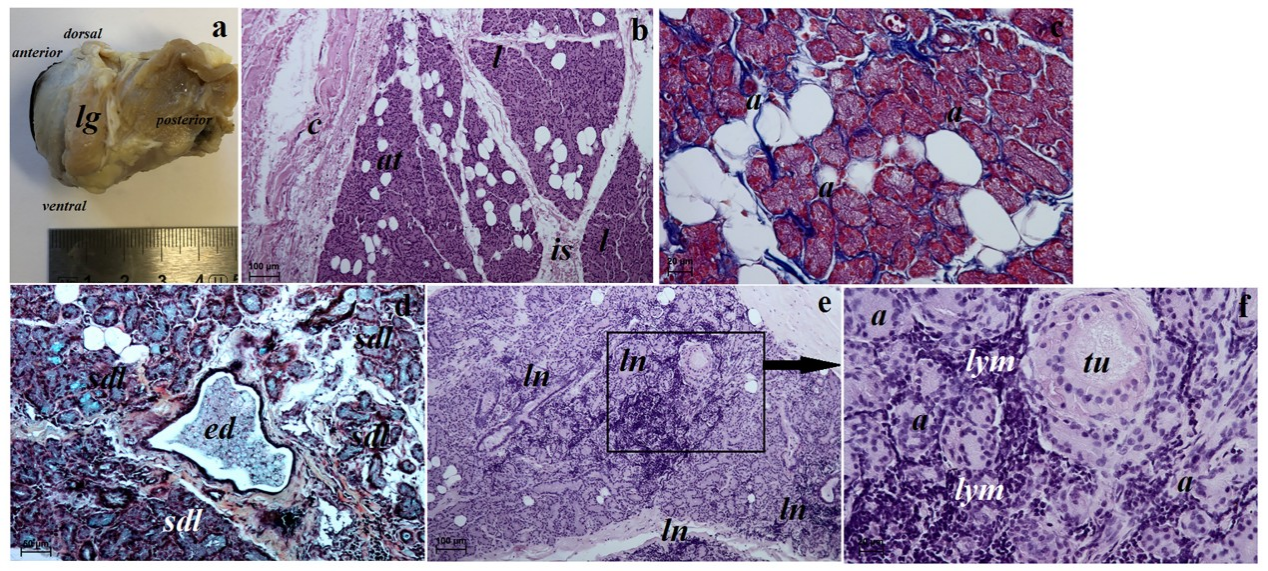
The macrograph (a) and histological images (b–f) of the lacrimal gland of the females South African Painted Dog. (a) view of the lacrimal gland (see *dorsal*, *ventral*, *anterior* and *posterior* orientation of the eyeball). (b, e, f) lacrimal gland. H&E staining. (c) lacrimal gland. Azan trichrome staining. (d) lacrimal gland. Movat pentachrome staining. *a*—acini, *at*—adipose tissue, *c*—capsule, *ed*—excretory duct, *is*—interlobar septa, *l*—lobes, *lg*—lacrimal gland, *ln*—lymph nodule, *lym*—lymphocytes, *sdl*—serous demilunes, *tu*—tubules. Scale bars: (a) = 5 cm, (b, e) = 100 μm, (d) = 50 μm, (c, f) = 20 μm.

The lacrimal gland in the examined females was triangular and light pink. It was located in the lateral canthus of the eye between the dorsal straight and lateral straight muscles in the dorsolateral angle of the periorbit (Fig 11a). The morphometric parameters of the lacrimal gland (length, width, thickness) in the two females can be found in Table 4. All values were comparable.

#### Histological and histochemical examination

**Fig 12:**
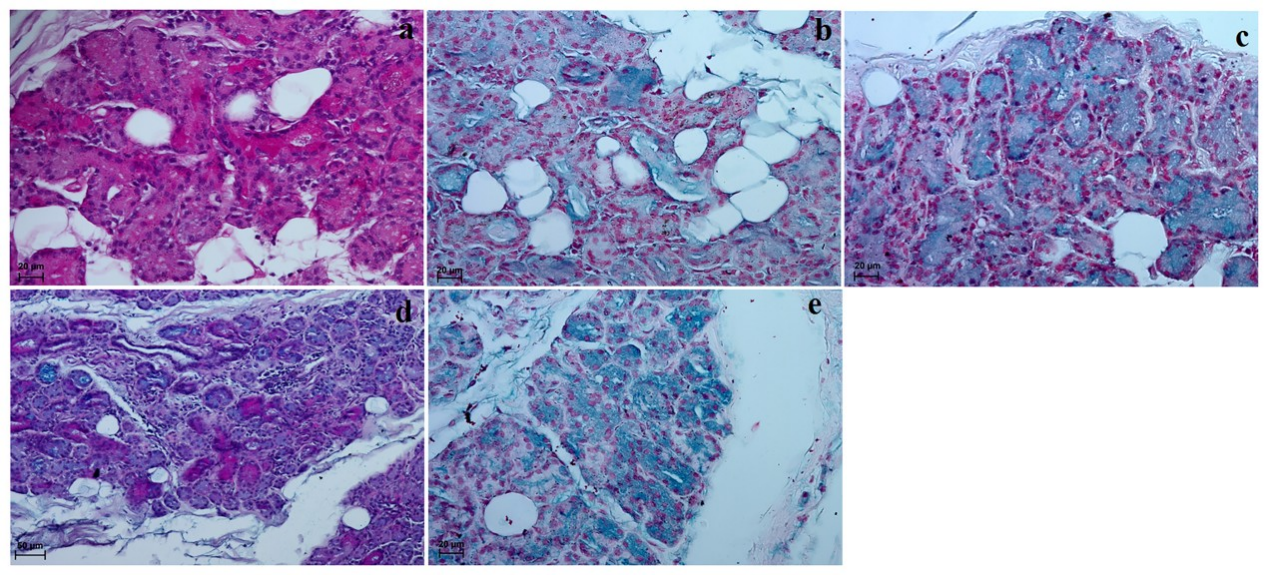
The histochemical images of the lacrimal gland of the females South African painted dog. (a) P.A.S. strongly positive reaction (+++) in the acini. (b) AB pH 1.0 weakly reaction (+) in the secretory cells. (c) AB pH 2.5 slightly reaction (+) in the glandular acini. (d) AB pH 2.5 P.A.S. strongly positive reaction (+++, violet color) in the acini. (e) HDI middle positive reaction (+/++) in the secretory cells. Scale bars: (a–c, e) = 20 μm, (d) = 50 μm.

The lacrimal gland in both females had a multilobar tubuloacinar structure producing a sero-mucous secretion (Table 6 and Fig 12). It was surrounded by a thin connective tissue capsule infiltrated with adipocyte aggregates, which formed a connective tissue septum dividing the gland into several large lobes and few small lobes (Fig 11b). This connective tissue consisted of collagen and elastic fibres, numerous adipocytes and blood vessels. The acini had a small lumen and consisted of tall conical secretory cells with a basophilic cytoplasm (Fig 11c). The tubules had cuboidal cells with an oval nucleus (Fig 11c). The numerous excretory ducts were lined by a simple cuboidal epithelium (Fig 11d). The Movat pentachrome staining revealed the presence of numerous serous demilunes with a moderately positive reaction (++) indicating a lymph node aggregates surrounding the acini and tubules were noted (Fig 11e and 11f). The measurements of the lacrimal gland are found in Table 5.

Histometric studies of the analyzed structures in the superficial gland of the third eyelid and lacrimal gland showed that the thickness of the superficial gland capsule was twice that of the lacrimal gland, while the thickness of interlobar septa and tubular outer diameter was slightly greater in the lacrimal gland than in the superficial gland of the eyelid.

## Discussion

The *Lycaon pictus* is currently the only representative of the *Lycaon* genus. It is a representative of relict African fauna once present in Eurasia. As evidenced by fossil records, lycaon ancestors were present in the Pliocene in Asia and Europe and in the Pleistocene (approximately 1 milion years ago) also in Africa and were very similar to modern wild dogs [36]. The *Lycaon pictus* was earlier placed in the *Simocyoninae* subfamily together with the dhole (*Cuon*) and the bush dog (*Speothos venaticus*). Recent studies of the mitochondrial DNA of the African wild dog showed a close relationship with canines (*Canidae*) of the *Canis*, *Cuon*, *Cerdocyon*, *Atelocynus*, *Atelocynus*, *Dusicyon* and *Lycalopex* genus [37]. According to Johnsing [38] and Venkataraman [39], the African wild dogs and dholes (*Cuon alpinus*) have a similar morphology, behavior and ecology. Radinsky [40] writes in his study that the South African Painted Dogs has the same number of chromosomes and similar neuroanatomy as the domestic dog (*Canis lupus familiaris*). Unfortunately there are fewer studies concerning the exact orbit and eye anatomy or studies in the fied of veterinary opthalmology in canines of the *Atelocynus*, *Cuon*, *Cerdocyon* [21, 41, 42], *Chrysocyon* [41, 43] *Dusicyon*, *Lycalopex*, *Nyctereutes*, *Otocyon*, *Speothos*, *Urocyon*, *and Vulpes genus* than of the *Canis* genus (and the *Canis lupus familairis* in particular). Hence, the presented results significantly expand the existing knowledge on comparative anatomy in the orbit, eye and chosen accessory organs in wild *Canidae*.

### The eyeball and eye tunics

The macroscopic measurements of the eyeball performed in the examined South African Painted Dogs were compared with those of the domestic dogs including mongrel dogs nad breed dogs present in literature [21, 44] and the crab-eating fox [21]. They showed that the female wild dog had a larger orbit than the crab-eating fox, mongrel dog and breed dog. This is most likely associated with craniometry, the type of skull and orbital dimensions, which, in the case of the domestic dog, are strictly associated with the animal’s size and are discussed further in this section.

The pupil in the examined South African Painted Dogs was round, similarly to that of the domestic dog [44], maned wolf (*Chrysocyon brachyurus*) [41] and gray wolf (*Canis lupus lupus*) [45]. It was vertically slit-shaped in the crab-eating fox and European red fox (*Vulpes vulpes*) [41, 42, 45]. Banks *et al*. [46] and Malmström and Kröger [45] suggested that the pupil shape and size is associated with an ecological niche occupied by a given species, hence its adaptation to multifocal optical systems and whether they are “predators” or “prey” (for herbivores (preys) animals, daytime predators and as in the nocturnal predators, diurnal-nocturnal predators and crepuscular vertebrates in order to reach maximum light-gathering ability). According to Castello [10] and Nyakatura and Bininda-Emonds [47] the South African Painted Dogs is predominantly the diurnal predators similar to the gray wolf, which displays the evening activity—after dusk and in the morning). Phylogenetic study (molecular data, with estimated divergence times) showed that the wolf-like canids include two species, however both of them live in a different ecological niche (the gray wolf inhabits of the forests, plains, marsh, Eurasia and North America mountains), while the South African Painted dog occupies the Angola, Bostwana, Malawi, Namibia, South Africa, Zambia, Zimbabwe areas) [10, 47]. On the other hand, the maned wolf belongs to the South American canids and is crepuscular or nocturnal predators, which rest during the day between of the tall grass and brake [10, 47].

Macroscopically, the *tapetum lucidum* in the female South African Painted dog was similar in shape to a semicircle and was milky. According to numerous authors, the *tapetum lucidum* was green in the crab-eating fox and domestic dog [21], while it was yellow with a green border in the crab-eating fox and maned wolf [41]. According to Ollivier *et al*. [47] it varies from yellow-green to green—blue with an irregular marginal area in the dog, while according to Murphy *et al*. [44] the *tapetum lucidum* in dogs was a rounded right trianle.

Histological studies revealed that the anterior corneal epthelium in the South African Painted Dogs females was covered by a non-keratinized stratified squamous epithelium consisting of 9–12 rows of cells. In the crab-eating fox there were four to five cell rows, while in the domestic dogs there were six to nine cell rows [42, 48]. In the *Canis lupus* there were 10 cellular layers, in the *Canis dingo* there were six to 8 cellular layers, while in the *Vulpex corsac* there were five to seven cell rows [49]. Nautscher et al. [48] showed that the number of cell rows has correlation with the corneal thickness. Additionally in the antral corneal loci the corneal epithelium was the thickest, while in the limbal loci was the thinnest [50].

Histologically, the *tapetum lucidum* in the examined femals was similar to that of other *Canidae* in that it was cellular (*tapetum lucidum cellulosum*) and was composed of elongated oval cells in its center, progressively thining and eventually disappearing towards the periphery. As described by Ollivier *et al*. [51], the tapetal cells in the domestic dogs formed a multiple layered “brick wall” where each cell was a rounded polygon. According to Yamaune *et al*. [52], the atypical *tapetum lucidum* was observed in aged dogs. The *tapetum lucidum* in the South African Painted Dogs composed multiple of the maximum 14–17 layers of the tapetal cells. As reported by Wen *et al*. [50] and Chijiiwa *et al*. [53], the canine *tapetum lucidum* has been reported to contain 9 to 11 layers of tapetal cells, while Lesiuk and Braekvelt [54] and Yamaue *et al*. [52] described 15 to 20 layers of cells.

### The bony orbit

A detailed anatomical description of the bony orbit in the South African Painted Dogs revealed that, similarly to other canines, it was an open orbit composed of the same bony structures as in the domestic dogs. However, there were also differences between this structure in the South African Painted Dogs and the domestic dog. These included the presence of a single ethmoid opening in the South African Painted Dogs versus a double opening in the domestic dog [35]. Furthermore, the pterygoid crest was more pronounced in the South African Painted Dogs than the domestic dog. The lacrimal sac, which was present in the domestic dog [35] was not palpated in the studied dogs. However, Evans [20] arguments that it is not always present in domestic dog skulls. Similarly, the groove for the ventral oblique ocular muscle was not found in the studied females, but has been reported in the domectic dog [35].

The orbital morphometric measurements (vertical length, horizontal width, orbital depth, orbital area, interorbital distances, and lengths of the frontal, lacrimal and malar) were compared between the South African Painted Dogs, the crab-eating fox and domestic dogs [21]. The measurements of the vertical length, horizontal width, orbital depth and interorbital distances were higher in the examined wild dogs than in in the domestic dogs and crab-eating fox. The orbital area was almost 2.5 times larger in the South African Painted Dogs than the remaining species, whereby it was comparable between the crab-eating fox and domestic dog. The frontal length was two times larger in the examined females than in the crab-eating fox and domestic dogs. The lacrimal length and frontal length were comparable to that of the domestic dogs, while it was almost two times smaller and 1.5 times smaller than in the crab-eating fox, respectively [21]. The orbital index in the female South African Painted Dog was 91.021 ± 2.08% and was higher than the orbital index in female 80.35 ± 9.102% and males 81.57 ± 4.295% Nigerian local dogs [55]. The orbital index in the crab-eating fox and the mongrel mesocephalic domestic dog did not take gender into account and amounted to 91.08% and 97.54%, respectively. Based on our results, the orbital index in the South African Painted Dogs was comparable to that of the crab-eating fox, while it was much lower than in the mongrel mesocephalic domestic dog [21]. As reported by Martinez *et al*. [56], the skull morphometry in the crab-eating fox features variations in anatomical composition, which is conditioned by the climate conditions and geographical adaptation of different populations. Similar observations were made with respect to the North American wild canis (*Canis lupus*, *Canis latrans*, *Canis rufus*) by Schmitt and Wallace [57]. By comparing the above described *Canis familiaris* genuses, Schmitt and Wallace [57] showed that the orbital dimesions were larger than in the wild canids. Such differences in the orbit measurements ale most likely associated with the skull type (dolichocephalic, mesocephalic and brachycephalic), which are defined in relation to domestic dogs on the basis of detailed craniometry taking into account the breed of dog or mongrel dogs [58–61]. An example is the study by Neto *et al*. [62], who qualified the skull type as mesocephalic in the crab-eating fox based on the skull measurements. Numerous studies on craniometry or skull morphology carried out by scientists significantly focused on the cranial ontogenesis and the relationship between the skull form and the developmental, ecological, and evolutionary aspects, although skull types have not been categorized in other *Canidae* [63–74]. This significantly limits correlation studies of the skull mophometry and the orbit morphometry performed by veterinarians specialized in veterinary ophthalmology of wild or captive animals. Considering the above, we cannot fully compare the obtained orbit measurements of the South African Painted Dogs with those of domestic dogs or other wild *Canidae*. We were limited by the studied population size and the fact that the dogs were only female, hence we did not perform a craniometry in the South African Painted Dogs, which we hope to do if we have access to a larger population of this species. If so, we would also like to perform computed tomography (CT) scans of the skull and ultrasound examinations of the eye.

Craniometric studies and measurements of the mandible collected by Zurano *et al*. [75] in the South African canids of the *Atelocynus*, *Cerdocyon* and *Lycalopex* genus with respect to the ecological divergence revealed that as “the species diverged, they evolved distinct climatic tolerances. Climatic niche similarities are not related to species phylo-genetic relationships, indicating that closely related species may have distinct climatic tolerances”. Zurano *et al*. [75] „suggest that these differences were related to climatic and trophic niches and ich results show divergent phenotypes in both the skull and mandibula, and that there is a close association between phenotype and ecological strategies”.

### The upper and lower eyelids

The macroscopic studies in the upper and lower eyelids in African wild dogs showed no eyelashes in the anterior palpebral margin in the lower eyelids, similarly to the domestic dog [76, 77] and crab-eating fox [21]. As reported by Carvalho *et al*. [41] short and scarce accessory eyelashes were found in the lower eyelid of the maned wolf and the crab-eating fox. The palpebral conjunctiva in the examined South African Painted Dogs was strongly pigemnted and was brown, similarly to the crab-eating fox [21]. Histologically, both eyelids were structurally similar but also differed. The tarsal glands were more developed in the lower eyelids (whose secretion formed part of the superficial oil layer of the tear film), similarly to the crab-eating fox and in contrast to the domestic dog [21]. As reported by Carvalho *et al*. [41], the tarsal glands in the maned wolf were developed equally. One to two large conjunctival folds which featured numerous goblet cells with a strong strongly P.A.S (for neutral glycoproteins, AB pH 1.0 (for acid sulphated glycoproteins), AB pH 2.5 (for acid sialylated glycoproteins), AB pH 2.5 P.A.S (for neutral and acid sialylated glycoproteins) and HDI (for sulfated acid mucopolysaccharydes *SAM* or carboxylated acid mucopolysaccharydes *CAM*) positive reaction and single melanocyte aggregates were found within the orbital zone. The density of goblet cells at bulbar conjunctiva differs between species and to the free-living condition, higher number of goblet cells implicates in higher mucin production and in ocular surface protection [78–80]. In domestic dog the highest density of the goblet cells was observed in the lower conjunctival fornix [78]. The presence of a single conjunctival lymphoid nodule aggregate located in the lymphoid region in lower eyelid was also found. In contrast, the presence of lymphatic cells in the lamina propria of the eyelids was found in the crab-eating fox [21]. It was not reported whether they were typical CALT conjunctival lymphoid nodule agregates, intraepithelial lymphocytes, subepithelial lymphoid cells or diffuse lympocytes adjacent lymphatic and blood vessels, were covered by follicle-associated epithelium (FAE) [81–83]. Characteristically, there are no goblet cells in the lymphoid region of the epithelium. However, sparse goblet cells in the bulbar cojnunctiva as well as in the third eyelid conjunctiva were reported in the crab-eating foxes and domestic dogs [21]. According to Wenzel-Hora *et al*. [84], the number, size, and location of the conjunctival lymph nodules vary with the age of the dog and the degree of antigenic stimulation. Our studies also found that the posterior surface of the eyelids was covered by a stratified columnar epithelium with varying numbers of cell layers in the marigal zone and orbital zone. In domestic dogs, this area is known as the palpebral cojnunctiva and is covered by a stratified squamous epithelium, while the bulbar conjunctiva located in the upper and lower conjunctival fornix was covered by a stratified cuboidal or columar epithelium [85].

### The superficial gland of the third eyelid and the third eyelid

Our research showed that similarly to the domestig dog and crab-eating fox [44, 86, 87], the superficial gland of the eyelid in the South African Painted Dogs was located in the medial canthus of the eye between the medial straight and ventral straight muscles. In the South African Painted Dogs, the gland was oval and light pink, while it was pink and tear-drop shaped in the domestic dog [44]. Our morphometric study found that the superficial gland of the third eyelid was longer and thinner in the female South African Painted Dogs than in the crab-eating fox [21] and domestic dogs [21] (in the latter two species the authors did not consider sexual dimorphism). The study by Cabral *et al*. [88] in mongrel dogs with sexual dimorphism showed that the superficial gland was thicker in males than in females, while there were no statistically significant differences in the length and width between sexes. When comparing our results with those of Carbal *et al*. [88], the gland in the South African Painted Dog was longer, wider and thicker than in the female mongrel dogs. Histologically, the superficial gland in the South African Painted Dogs had a multilobar tubuloacinar structure. The histochemical study revealed that it was P.A.S—weakly positive (for neutral glycoproteins), AB pH 1.0—slightly positive (for acid sulphated glycoproteins), AB pH 2.5—middle positive (for acid sialylated glycoproteins), AB pH 2.5 P.A.S—weakly positive (for neutral and acid sialylated glycoproteins) and HDI—strongly positive (for sulfated acid mucopolysaccharydes *SAM* or carboxylated acid mucopolysaccharydes *CAM*). When using the Movat-pentachrome stain (single mucoserous units), it was found that the gland produced a serous secretion comprising an aqueous layers of the tear film. According to Cazacu [86] and Murphy *et al*. [44], this gland is also tubuloacinar seromucous gland in the domestic dog.

The third eyelid in the examined female wild dogs was T-shaped, similarly to previously described in other *Canidae* and was located in the medial canthus of the eye [21, 41, 44, 75, 85–87, 89]. Our study found that the free margin in the third eyelid in the African wild dogs was strongly pigmented, similarly to the crab-eating fox and the maned wolf [21, 41]. The morphometric study of the third eyelid performed on anthe examined African wild dogs and by Lantyer-Araujo *et al*. [21] in the crab-eating foxes and domestic dogs revealed that the third eyelid was longer in the mongrel mesocephalic domestic dogs than the African wild dogs and crab-eating fox. This study also revealed that it was T-shaped in the African domestic dog and the crab-eating fox [21, 44, 76]. According to Saito *et al*. [90], scanning electron microscopy reveaed that surgical removal of the third eyelid in dogs caused large morphologic changes in the corneal epithelium including decreased bright cell exfoliation, intercellular detachment of superficial cell layers and hemidesmosome detachment of basal cell layers. The histological study in the examined South African Painted Dogs revealed that the cartilage of the third eyelid was surrounded by thick layers of collagen and elastic fibres and was composed of hyaline tissue, similarly to the domestic dog or crab-eating fox [21, 41, 76]. We observed three conjunctival lymph nodule aggregates within the bulbar conjunctiva of the third eyelid, which was also seen in the domestic dog [76], or a single aggregate in the crab-eating fox [21]. As described by Constantinescu and Moore [76] the prominent conjunctival lymphonoduli aggregate gives a cobblestone appearance to the conjunctiva.

Cherry eye is often diagnosed in domestic dogs. This disorder is associated with the prolapse of the superficial gland of the third eyelid. This prolapse is usually deep red, round and can reach the size of a cherry. The cause of the disorder is thought to originate in the abnormality of the connective tissue attachments between the third eyelid and the periorbita [76]. If the gland is not reposited within a short period of time, the inflammatory state can damage the secretory functions of the lacrimal gland [76].

### The lacrimal gland

In our study, the lacrimal gland of the South African Painted Dogs was located in the lateral canthus of the eye between the dorsal straight and lateral straight muscles in the dorsolateral angle of the periorbital located, which is similar other reported *Canidae* [21, 44, 87, 90–92]. It was triangular and light pink. According to El-naseery *et al*. [91], Park *et al*. [87] and Zwingenberger *et al*. [93] the lacrimal gland in the domestic dogs was flatened, pink and varied in shape, showing morphological variations between domestic dogs, differing due to breed (oval, round, rectangular, triangular, heart—or dumbell—shaped). The morphometric analysis of the lacrimal gland in the wild *Canidae* in our study revealed that it was almost twice as long and comparably wide as in the crab-eating fox. When comparing these results to domestic dogs, the breed, sex and measurements of the mongrel dogs as well as the skull type should be taken into consideration, as they may impact the morphometric differences of this gland [21, 87, 90, 91]. Histological and histochemical analyses in the wild dogs revealed that this gland had a tubuloacinar structure with numerous serous demilunes and a moderately positive reaction in the Movat pentachrome staining, which produced a sero-mucous secretion (P.A.S strongly positive reaction (+++), AB pH 1.0 weak reaction (+), AB pH 2.5 slight reaction (+), AB pH 2.5 P.A.S. strongly positive reaction (+++) and HDI moderately positive reaction (+/++)) similarly to domestic dogs [44, 90, 91, 93, 94]. The performed histological study in the older of the two South African Painted Dogs showed the presence of numerous large lymphnodule aggregates in the connective tissue surrounding the acini and tubules, which were not observed in the younger female. Other than an age-related difference, we assume that this may have been associated with an inflammatoru process within the lacrimal gland. However, neither the caregivers or veterinarians nor the *post mortem* findings indicated any inflammatory process in the eye, such as ocular discharge sticking the eyelids together, dirty eyes, redness of the conjunctiva or swelling.

### The precorneal tear film

The precorneal tear film performs many important functions ensuring the correct functioning of the eye. These include maintaining a smooth surface for light refraction, lubrictaing the upper and lower eyelids, conjunctiva and cornea, suppyling the cornea with nutrients and transporting metabolic by—products from the corenal surface, providing leucocytes wtih access to the cornea and conjunctiva, removing foreign materials from the conjunctiva and cornea, defending the ocular surface from pathogens via specific and nonspecific antibacterial substances [81, 95]. The tear film consists of the three layers: a superficial oily layer, a central aqueous (serous) layer, and a thin mucous (glycoproteinaceous) layer covering the cornea. The first layer is oily and is produced by the tarsal glands, which are commonly known as Meibomian glands. They provide lubrication and a smooth optical surface, they prevent overflow of tears from the lid margins, reduce the evaporation of the underlying aqueous layer (tears) and prevent contamination of the precornael tear film from debris [44, 81]. The second layer is the aqueous layer and is a major component of the precorneal tear film. It is understood to mix readily with the subjacent mucous later. It is produced by the lacrimal gland and the superficial gland of the third eyelid as well as by the accessory lacrimal glands (Krause’s glands and Wolfring’s glands) located in the upper and lower eye lids. It contains a variety of factors necessary for maintaining corneal health. The aqueous layer also contains antimicrobial compounds such as transferrin and the immunoglobulin IgA, IgG, and IgM, albumin, ceruloplasmim, tear specific prealbumin, glycoprtiens with the participate in the defense of the ocular surface [95, 96]. The third layer is the glycoproteinaceous or mucous layer, which is intimately associated with the surface of the superficial squamous cells of the anterior corneal epithelium. This layer is produced by the goblet cells located in the palpebral conjunctiva and is thought to assist in the adherence of the precorneal tear film to the corneal surface by decreasing the surface tension of the tears [44, 97]. It is composed of mucin, immunoglobulins, urea, salts, glucose, leukocytes, cellular debris and enzymes [98]. Any quantitative or qualitative abnormalities of the three layers in the tear film are associated with a disease process within the lacrimal system, eyelids and orbital glands. As a result, they may lead to corneal lesions or chronic inflammatory disease such as *keratoconjunctivitis sicca* (*KCS*) (dry eye) frequently diagnosed in domestic dogs [99, 100].

This study is part of a series of publications constituting the PhD thesis of Wojciech Paszta DVM supervised by Prof. Joanna Klećkowska-Nawrot PhD DVM and Karolina Goździewska-Harłajczuk PhD DVM.

## Supporting information

S1 Fig Skulls of the two females (1 and 2) of the South African painted dog (*Lycaon pictus pictus*). (a) female 1—right side; (b) female 1—left side; (c) female 2—right side; (d) female 2—left side; (e)—female 1 and 2.
(TIF)
