# Supplementary figures and images for: Anatomical and morphometric evaluation of the orbit, eye tunics, eyelids and orbital glands of the captive females of the South African painted dog (Lycaon pictus pictus Temminck, 1820) (Caniformia: Canidae)
Source: PLoS One. 2021 Apr 19;16(4):e0249368. doi: 10.1371/journal.pone.0249368 (PMC8055035; doi:10.1371/journal.pone.0249368)

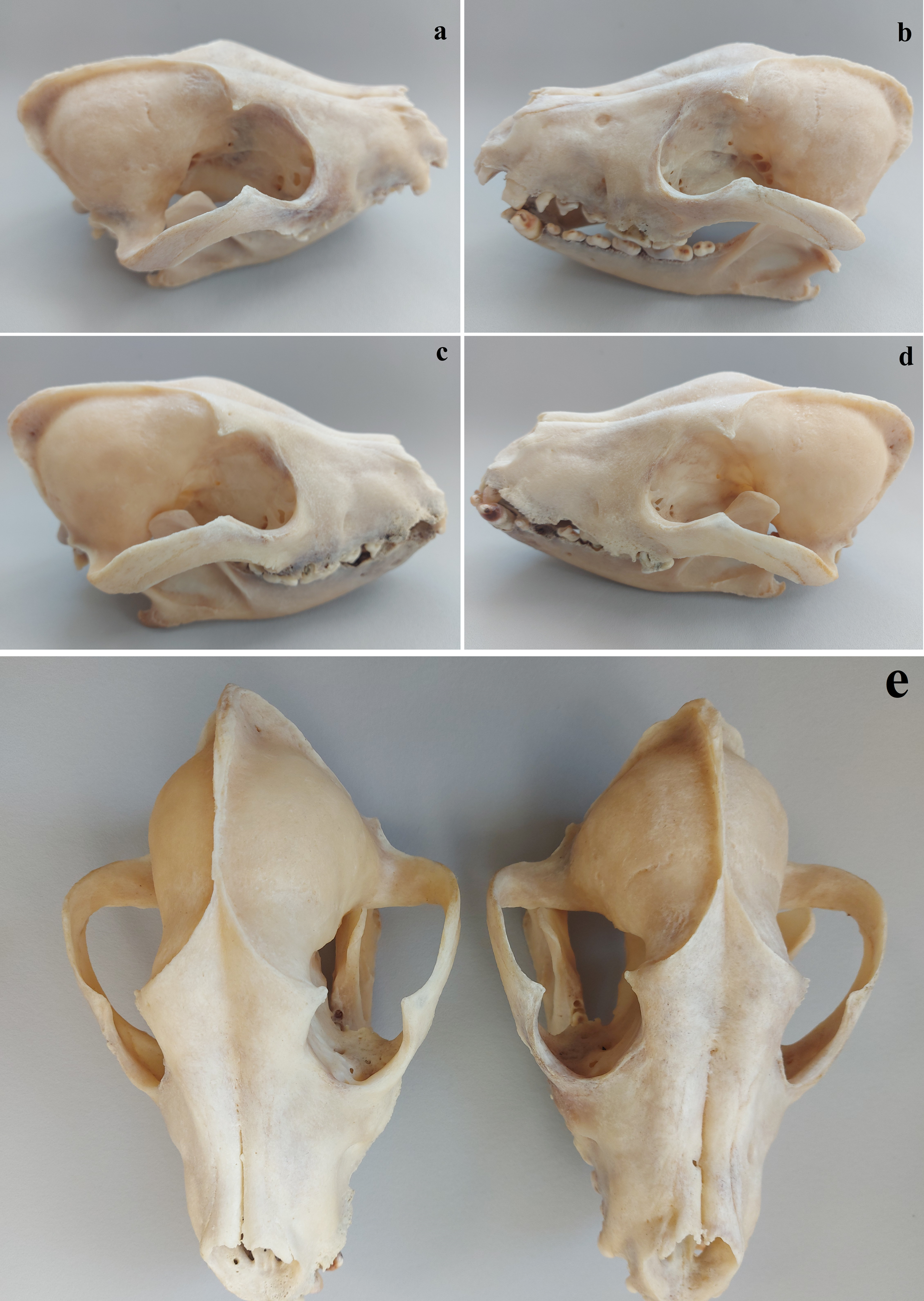

Supplement: S1 Fig — (a) female 1—right side; (b) female 1—left side; (c) female 2—right side; (d) female 2—left side; (e)—female 1 and 2. (TIF) [file pone.0249368.s001.tif]
